# Dishevelled Paralogs in Vertebrate Development: Redundant or Distinct?

**DOI:** 10.3389/fcell.2017.00059

**Published:** 2017-05-26

**Authors:** Marc Gentzel, Alexandra Schambony

**Affiliations:** ^1^Molecular Analysis—Mass Spectrometry, Center for Molecular and Cellular Bioengineering (CMCB), TU DresdenDresden, Germany; ^2^Developmental Biology, Biology Department, Friedrich-Alexander University Erlangen-NurembergErlangen, Germany

**Keywords:** Dishevelled, Wnt signaling, vertebrate embryonic development, embryonic expression, autosomal dominant robinow syndrome

## Abstract

Dishevelled (DVL) proteins are highly conserved in the animal kingdom and are important key players in β-Catenin-dependent and -independent Wnt signaling pathways. Vertebrate genomes typically comprise three DVL genes, DVL1, DVL2, and DVL3. Expression patterns and developmental functions of the three vertebrate DVL proteins however, are only partially redundant in any given species. Moreover, expression and function of DVL isoforms have diverged between different vertebrate species. All DVL proteins share basic functionality in Wnt signal transduction. Additional, paralog-specific interactions and functions combined with context-dependent availability of DVL isoforms may play a central role in defining Wnt signaling specificity and add selectivity toward distinct downstream pathways. In this review, we recapitulate briefly cellular functions of DVL paralogs, their role in vertebrate embryonic development and congenital disease.

## Introduction

The Dishevelled (dsh^1^) phenotype has been described the first time in Drosophila close to 60 years ago (Fahmy and Fahmy, 1959) and the diverse molecular functions of Dishevelled (DVL) proteins still stimulate intensive research. To date DVL is considered the central intracellular effector of Wnt signaling pathways, which play key roles in establishing and patterning of the body axes and in the control of proliferation, differentiation, planar cell polarity (PCP), and cell movements throughout the animal kingdom. Although evidence is accumulating that Wnt pathways should be considered as a signaling network, individual pathways have been subdivided into the Wnt/β-Catenin pathway and the β-Catenin–independent pathways including Wnt/PCP, Wnt/Ca^2+^, and Wnt/STOP signaling, all of which involve DVL (reviewed in Kühl et al., 2000; Kohn and Moon, 2005; Macdonald et al., 2009; Niehrs and Acebron, 2012; van Amerongen, 2012; Collu and Mlodzik, 2015, Figure 1A).

**Figure 1:**
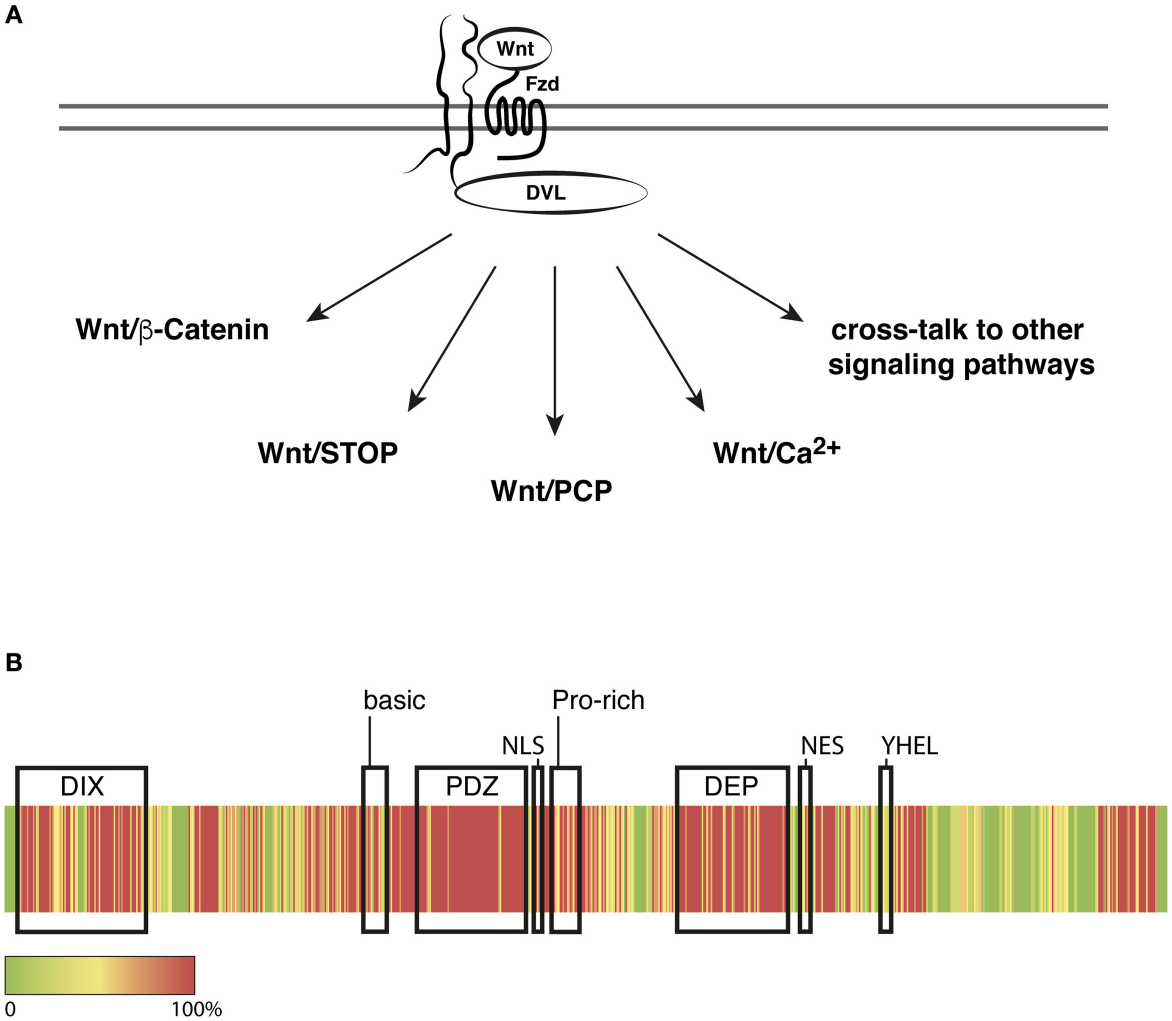
**Dishevelled in Wnt/Frizzled signaling. (A)** DVL relays Wnt/Frizzled signals to multiple signaling pathways to regulate cellular functions including mitosis, transcription, polarity, and migration. **(B)** Heatmap representation of the conservation of all three DVL proteins in human, rat, mouse, and frog. Sequence alignments were calculated using Clustal Omega with the following input sequences: *Homo sapiens* DVL1: O14640, DVL2: O14641, DVL3: Q92997, *Rattus norvegicus* DVL1: Q9WVB9, DVL2: D3ZB71, DVL3: D4ADV8, *Mus musculus* DVL1: P51141, DVL2: Q60838, DVL3: Q61062, *Xenopus laevis* DVL2: P51142, DVL3: Q6DKE2 (Uniprot Accession numbers), *Xenopus laevis* DVL1: NCBI XP_018081523. Red indicates 100% identity and Green indicates no identity of amino acids at the respective position. Conservation scores were calculated according to Livingstone and Barton (1993). Functional domains or motifs are indicated by correspondingly labeled boxes; for details and references see text.

DVL and its core functions in β-Catenin-dependent and -independent Wnt pathways are highly conserved. Notably, the genomes of Drosophila and most other invertebrates harbor only a single *DVL* gene. By contrast vertebrate genomes comprise genes for three *DVL* paralogs (*DVL1-3*), which have most likely arisen from two rounds of genome duplication (reviewed in Kasahara, 2007; Dillman et al., 2013). The still high degree of conservation among the three vertebrate DVL paralogs raises the question to which extent they have undergone functional diversification since obviously a single protein is sufficient to mediate DVL functions in invertebrates.

## Cellular functions of dishevelled paralogs

All DVL proteins share an N-terminal DIX domain, a PDZ and a DEP domain (Axelrod et al., 1998; Boutros et al., 1998; Li et al., 1999). Additional sequence motifs have been identified that provide interfaces for protein-protein interactions like the basic and proline-rich regions, or functional motifs as the nuclear export sequence (NES), a putative nuclear localization sequence (NLS), the—YHEL—motif that is required for internalization of the activated receptor complex and motifs that mediate the association of DVL with the cytoskeleton or with phospholipids (Capelluto et al., 2002; Penton et al., 2002; Itoh et al., 2005; Yu et al., 2007, Figure 1B). The lack of striking differences in the primary structure with no obvious additional or missing functional motifs and domains poses a challenge to predict functional differences and to develop assays that would reveal potential dissimilarities.

DVL interacts with the cytoplasmic interface of Frizzled receptors (Tauriello et al., 2012), regulates receptor internalization and maintenance of Frizzled membrane equilibrium through protein-protein interaction (Yu et al., 2007; Jiang et al., 2015) and serves as scaffold for numerous proteins including multiple protein kinases (reviewed in Gao and Chen, 2010; Mlodzik, 2016). Depending on the composition of the receptor complex and its interaction partners, DVL contributes to β-Catenin stabilization, inhibition of GSK3β or activation of β-Catenin-independent signaling cascades (Gao and Chen, 2010). DVL also enters the nucleus and interacts with TCF/c-Jun/β-Catenin or FOXK1/2 transcription factor complexes (Itoh et al., 2005; Gan et al., 2008; Wang et al., 2015). In addition, DVL proteins play a role in microtubule stabilization (Ciani et al., 2004), centrosome positioning and separation (Sepich et al., 2011; Cervenka et al., 2016) and mediate cross-talk with other signaling pathways such as Notch or NF-κB (Deng et al., 2010; Collu et al., 2012).

Notably, for most of the abovementioned DVL functions no preference for a DVL paralog has been detected, although some studies suggest dose-dependent effects (e.g., Cervenka et al., 2016). A different picture was observed for the role of DVL paralogs in PCP in ciliated cells, which is required to position the basal bodies. DVL1 was required for intact PCP signaling and localized asymmetrically in multiciliated cells in the epidermis of *Xenopus* tadpoles. DVL2 was concentrated in dots in vicinity to the basal bodies that led to a local concentration of active RhoA and was required for basal body positioning. Localization of DVL2 itself was mediated by Inturned and according to current knowledge neither of both proteins plays a role in ciliogenesis in the fly (Park et al., 2008). In the mouse node, DVL2 and DVL3 were apically localized and polarized to the posterior side. Positioning of the basal body and directional flow was disturbed or absent in DVL1/DVL2 or DVL1/DVL3 deficient embryos (Hashimoto et al., 2010). Moreover, polarized localization of DVL, planar polarity of basal bodies and their docking could be separated experimentally although the detailed mechanism remains elusive (Park et al., 2008; Vladar et al., 2009; Hashimoto et al., 2010).

## DVL paralogs in vertebrate embryonic development

### Embryonic expression patterns

In early *Xenopus* embryos, *DVL2* and *DVL3* are present maternally, whereas *DVL1* expression is upregulated after the mid-blastula transition (Tadjuidje et al., 2011). At early gastrula stages, all three *DVL* paralogs are expressed in the prospective mesoderm including Spemann's organizer and, although weaker, in the ectoderm. Post-gastrula expression of *DVL1* and *DVL2* largely overlaps and is strongest in the neural tube, premigratory and migrating neural crest, as well as in the otic placode, the presomitic and somitic mesoderm. Notably, *DVL3* was not expressed in the neuroectoderm but restricted to the paraxial mesoderm, the heart, cranial placodes, and at tadpole stages to the branchial arches (Gray et al., 2009).

In chicken embryos, only two *DVL* genes, *DVL1* and *DVL3*, were identified. *DVL3* was already expressed in day 2 embryos and showed broad expression in most embryonic tissues whereas *DVL1* was upregulated only after day 2, i.e., after completion of neurogenesis, and showed a spatially restricted expression in the brain, strongest in the optic stalk, the olfactory bulb, and the ventral forebrain and spinal chord (Gray et al., 2009).

All murine *DVL* genes are maternally expressed in mouse oocytes and pre-implantation embryos, but interestingly individual protein levels differ considerably and dynamically from oocyte to blastocyst (Na et al., 2007). Post-implantation, *DVL1* expression was detected in the mesoderm, but not in the node, at stage E7.5. Post-gastrula, *DVL1* was expressed strongest in the neuroectoderm and later in the CNS, cranial and dorsal root ganglia, somites, the liver, kidney, intestine, and lung (Sussman et al., 1994). For *DVL2*, ubiquitous expression has been reported during embryogenesis in the mouse (Klingensmith et al., 1996). At E 7.5, *DVL3* has also been detected ubiquitously, but shortly after showed elevated expression in the CNS and the somitic mesoderm, the notochord, heart, dorsal root ganglia, and branchial arches and in the limb buds (Tsang et al., 1996; Diez-Roux et al., 2011).

Phylogenetic analyses suggest that *DVL1* separated first from the common ancestor of *DVL2/3*, which split into *DVL2* and *DVL3* in a second round of duplication (Gray et al., 2009). Consistent with corresponding functional divergence, expression of at least one *DVL2/3* paralog in the oocyte and pre-blastula embryo and of *DLV1* in the central nervous system appear to be conserved among vertebrates. Except for these conserved patterns, developmental expression of the three *DVL* genes is highly divergent within a species and among different species with *DVL2* expression showing the highest variability.

### Developmental function and human congenital disease

Both, animal models and human congenital disease provide insights into the developmental function of vertebrate DVL paralogs. To date, DVL has predominantly been studied in the mouse and, to a much lesser extent, in *Xenopus*. Transgenic, single, and compound knock-out mouse models have been discussed comprehensively and in detail in a review by Wynshaw-Boris (2012, see also Supplementary Table 1), therefore we will focus here on human congenital disease and related phenotypes in animal models. In humans, mutations in *DVL* genes have been associated with neural tube closure defects (NTD) and autosomal-dominant Robinow Syndrome (ADRS) (De Marco et al., 2013; Bunn et al., 2015; White et al., 2015, 2016).

#### Neural tube defects

**Figure 2:**
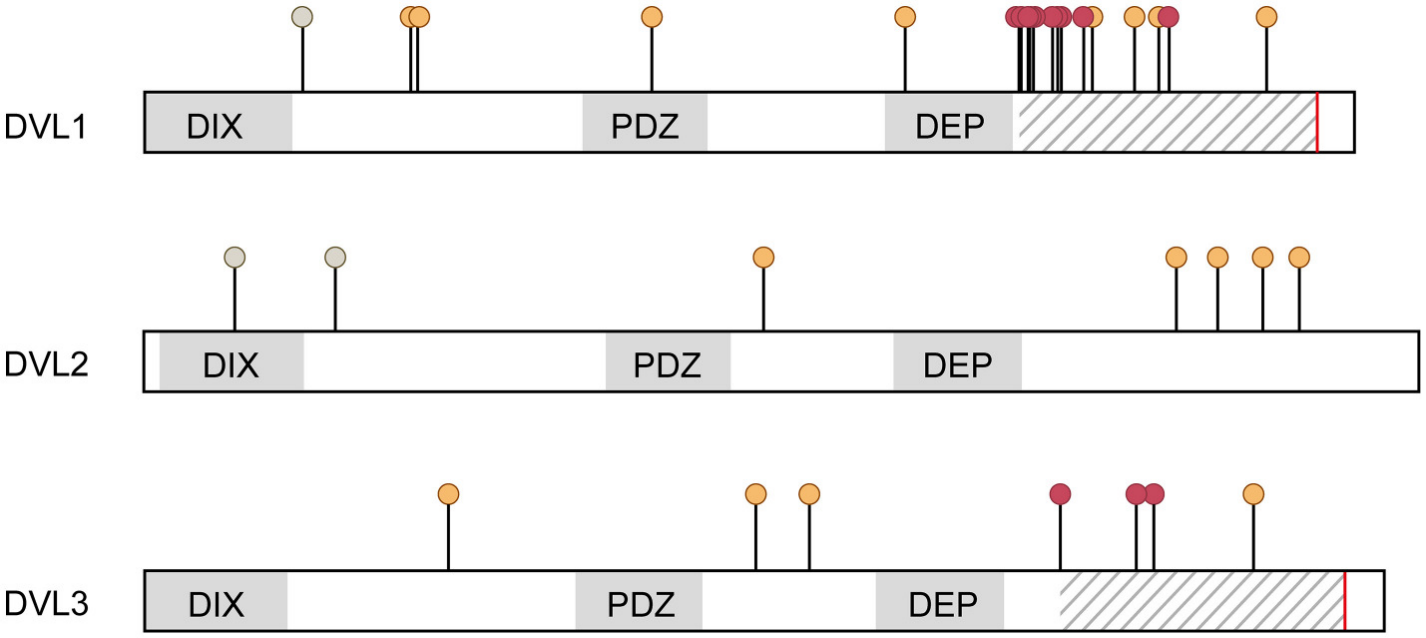
**Mutations identified in the three ***DVL*** genes in humans**. Mutations are indicated at the positions of amino acid changes. Changes detected in individuals with NTDs (De Marco et al., 2013; Merello et al., 2013; Chen et al., 2016) are color coded orange (predicted pathogenic) and gray (predicted benign, in all cases A>V). All ADRS mutations are (−1)-frameshift mutations resulting in altered amino acid sequences in the C-terminus and a premature stop (Bunn et al., 2015; White et al., 2015, 2016), which are indicated by hatched area and red line respectively. Positions of individual mutations associated with ADRS are labelled with red dots.

During embryonic development, the neural tube is formed by elevation, convergence and fusion of the lateral neural folds to form a hollow tube. Morphogenesis and closure of the neural tube is affected by nutritional, environmental and genetic factors including Wnt/PCP signaling, which is illustrated by genetic association between NTD in humans and mutations in the PCP genes *VANGL1, VANGL2, CELSR1, FZD6*, and *DVL2* (Cai and Shi, 2014; reviewed in De Marco et al., 2014). Notably, also mutations in *DVL1* or *DVL3* have been identified in humans with NTD (Figure 2), although the correlation was not significant (De Marco et al., 2013; Merello et al., 2013; Chen et al., 2016).

Consistently, neural tube closure also requires the same PCP factors in mouse, frog, and zebrafish (Darken et al., 2002; Hamblet et al., 2002; Jessen et al., 2002; Curtin et al., 2003; Formstone and Mason, 2005; Wang et al., 2006). DVL2^−/−^ mice show NTD while single and compound DVL1 and DVL3 deficient mice do not, suggesting that among the three *DVL* genes, *DVL2* is necessary and sufficient to mediate neural tube closure. However, DVL2^−/−^ DVL3^+/−^ and DVL1^−/−^ DVL2^−/−^ mice display much more severe NTD than DVL2^−/−^ mice (Hamblet et al., 2002; Wang et al., 2006; Etheridge et al., 2008), which strongly suggests that DVL1 and DVL3 contribute directly or indirectly to neural tube closure. Along this line, maternal single knock-down of either DVL2 or DVL3 in *Xenopus* caused NTD (Tadjuidje et al., 2011), supporting a contribution of DVL2 and DVL3.

#### Autosomal-dominant robinow syndrome

Robinow syndrome is a rare genetic disorder characterized by mesomelic limb shortening, short stature, cranio-facial malformations, microgenitalia, and occasional cardiac outflow tract defects with either autosomal dominant or recessive inheritance (reviewed in Robinow et al., 1969; Patton and Afzal, 2002). Notably and despite the multifaceted clinical presentation of affected individuals, NTD have neither been described in ADRS nor the more severe recessive Robinow syndrome (RRS). Two recent studies have identified mutations in exon 14 of *DVL1* and *DVL3* as causative for ADRS (Bunn et al., 2015; White et al., 2015, 2016, Figure 2). In addition, ADRS is also associated with *WNT5A* whereas RRS is caused by loss-of-function mutations in *ROR2* (Afzal et al., 2000; van Bokhoven et al., 2000; Person et al., 2010). Thus, the features of Robinow syndrome are generally considered as consequences of defective WNT5A/ROR2-mediated PCP signaling in multiple tissues (Wang et al., 2011) and are partially recapitulated in DVL deficient animal models.

Short stature and defects of the axial skeleton are likely related to impaired convergent extension movements of the paraxial mesoderm and defects in somitogenesis, which have also been reported for DVL2^−/−^, DVL1^−/−^;DVL2^−/−^, and DVL2^+/−^;DVL3^−/−^ mice as well as for *Xenopus* embryos deficient of any DVL paralog (Hamblet et al., 2002; Etheridge et al., 2008; Gray et al., 2009; Gentzel et al., 2015), indicating that all DVL paralogs contribute to the development of the axial skeleton although DVL2 seems of particular importance.

The characteristic cranio-facial deformations seen in ADRS or RRS indicate defective development of the neural crest (NC), which gives rise to the majority of cranial cartilage and bone. In addition, a subpopulation of the NC contributes to the cardiac outflow tract (OFT). Cranio-facial malformations are also visible in DVL1 and DVL2 morphant *Xenopus* embryos and the abovementioned mice. The latter and additionally DVL3^−/−^ animals also show cardiac OFT defects. In *Xenopus*, DVL1 or DVL2 morphant embryos showed normal NC induction but defects in NC migration. The NC is present in DVL3^−/−^ mice whereas the cardiac NC markers PITX2 and PLEXINA2 were decreased in mice lacking DVL2 (Hamblet et al., 2002; Etheridge et al., 2008; Gray et al., 2009), indicating differential roles of DVL2 and DVL3 in NC and cardiac development.

Interestingly, *DVL1* mutations in humans affect predominantly cranio-facial development with little or no aberrations in body height, whereas in *DVL3* and *WNT5A* associated ADRS craniofacial malformations are accompanied by short stature (Person et al., 2010; Bunn et al., 2015; White et al., 2015, 2016). In mouse, *DVL1* is predominantly expressed in the neuroectoderm (Sussman et al., 1994) and as discussed above, knock-out models suggest dominant roles of DVL2 and DVL3 in the development of the axial skeleton and the heart. Although the spatial expression of the three *DVL* isoforms in human embryos is unknown, the differential prevalence of defects in the axial skeleton in DVL1 and DVL3 associated ADRS supports a prevailing role of DVL2/3 in the paraxial mesoderm in mammals.

#### DVL signaling in embryonic development

The recently characterized mutations in *DVL1* and *DVL3* are frameshift mutations, which alter and shorten the C-termini in the corresponding proteins (White et al., 2015, 2016). This C-terminal domain has been shown to interact with ROR2, a major receptor in β-Catenin independent Wnt signaling and affected in RRS, and is required for ROR2-mediated inhibition of Wnt/β-Catenin signaling (Witte et al., 2010). An initial study suggests a gain of Wnt/β-Catenin activity in ADRS (Bunn et al., 2015), thus it is conceivable that ADRS mutations in *DVL1* and *DVL3* might result in defective β-Catenin independent Wnt signaling and concomitantly upregulate Wnt/β-Catenin activity.

Malformations of the axial skeleton seen in DVL2^−/−^ and DVL1^−/−^;DVL2^−/−^ mice are reminiscent of the phenotypes in ROR2 or WNT5A deficient embryos that can be attributed to aberrant PCP signaling in the paraxial mesoderm (reviewed in Stricker et al., 2017). Genetic interactions between DVL2 and DVL3 with VANGL2 in the mouse further suggest that DVL2 acts in Wnt/PCP signaling in neural tube closure and in cochlea development (Wang et al., 2006; Etheridge et al., 2008). In addition, NTDs and OFT defects in DVL1^−/−^;DVL2^−/−^ mice were similar to defects in VANGL2 mutants and rescued by a DVL2ΔDIX transgene, which is defective in β-Catenin signaling but retains activity in PCP signaling (Wang et al., 2006; Sinha et al., 2012). Notably, also DVL3 KO mice develop OFT defects, but no genetic interaction with VANGL2 in OFT morphogenesis could be demonstrated, indicating a non-redundant function of DVL3 (Etheridge et al., 2008).

Wnt/β-Catenin signaling also contributes to the development of the paraxial mesoderm, heart and neural crest, and patterns the neural tube. However, defective Wnt/β-Catenin signaling results in patterning defects of the dorsal mesoderm and affects proliferation, expansion, or specification of dorsal neural tube progenitors and neural crest (NC) cells (Greco et al., 1996; Ikeya et al., 1997; Pinson et al., 2000; Lou et al., 2008; Seldin et al., 2008; Valenta et al., 2011). By contrast, in either single or compound DVL knock-out mice, Wnt/β-Catenin signaling, and early dorsal mesoderm markers were unaffected. Only in triple knock-out mice a marked downregulation of Wnt/β-catenin signaling has been observed (Etheridge et al., 2008; Hashimoto et al., 2010). Still, defective β-Catenin signaling in smaller cell populations cannot be excluded. One such example might be the cardiac neural crest in DVL2^−/−^ embryos, in which the β-Catenin target PITX2 is downregulated (Hamblet et al., 2002; Kioussi et al., 2002). PITX2 is required for proliferation of cardiac NC, but also for the interaction between cardiac NC and second heart field cells (Kioussi et al., 2002; Ma et al., 2013) and, indirectly, for OFT morphogenesis via its target gene *WNT11* (Zhou et al., 2007). OFT defects in DLV1^−/−^, DVL2^−/−^ embryos were rescued by a DVL2ΔDIX transgene (Sinha et al., 2012), however this does not exclude a role of DVL2 in β-Catenin signaling upstream of PITX2 since the transgene could also restore OFT morphogenesis downstream of WNT11.

Overall it appears that DVL function in β-Catenin-independent Wnt signaling is more sensitive to the loss or dysfunction of one or two paralogs and accounts for most of the developmental phenotypes in knock-out animal models and also for the features of ADRS.

#### Induced heart defects

A number of studies indicate a specific role of DVL1 in cardiac remodeling and regeneration. DVL1 and CamKII are upregulated after induced myocardial infarction and heart failure indicating a role of Wnt/Ca^2+^ signaling in regeneration (Chen et al., 2004; Ai et al., 2005; Bossuyt et al., 2008). Persistent pressure overload induced cardiac hypertrophy was attenuated in *DVL1* knock-out mice, which has been attributed to lower Wnt/β-Catenin activity as well as decreased AKT activation (van de Schans et al., 2007). Consistently, DVL1 gain-of-function induced progressive cardiomyopathy (Malekar et al., 2010). Interestingly, evidence is accumulating that DVL1 is functionally associated with Wnt/Ca^2+^ and CamKII signaling in cardiomyopathy (Malekar et al., 2010; Zhang et al., 2015), in excitory synapses in the rat spinal chord (Ciani et al., 2011) and in convergent extension movements in *Xenopus* gastrulation (Gentzel et al., 2015), indicating a functional specification of DVL1.

## Perspectives

Striking differences between different DVLs and species have been observed in temporal and spatial expression patterns. Loss-of-function phenotypes of each single paralog in mouse as well as ADRS features associated with *DVL1* or *DVL3* mutations also differ, indicating some degree of divergence but also overlapping functions. In addition, expression of transgenes in a single knock-out background further supported partial redundancy and indicated a dose dependency. If the DVL paralogs would be fully redundant in function it might be speculated that the summed abundance of all paralogs is important for cell survival. But even in cell culture models, any single knock-down is effective and the cells apparently do not sense overall “DVL concentration” and do not compensate the down-regulation of one protein by upregulation of the others (Cervenka et al., 2016).

Functional redundancies however, do not connote biochemical identity. The observed differences could reflect differential expression levels, epistatic relations, or differential biochemical properties such as protein-protein interaction affinity and consequently also molecular function, which would also be consistent with dose-dependencies. The developmental phenotypes further indicate that β-Catenin independent Wnt pathways are more sensitive to the dose of individual DVL paralogs than β-Catenin signaling. This hypothesis is further supported by comparison of triple knock-out and triple-RNAi knock-down embryos. Whereas, in triple KO embryos early β-Catenin signaling is strongly reduced resulting in axis and mesodermal mispatterning, in the knock-down, in which ~25–30% of each paralog were retained, early β-Catenin signaling was not affected, but the embryos showed strong morphogenetic defects in the dorsal mesoderm and neuroectoderm. Consistently, specific and different molecular functionality of the three DVL paralogs has been observed in ciliogenesis and the Wnt/Ca^2+^ pathway, which were revealed in intact tissue or tissue models (Park et al., 2008; Gentzel et al., 2015).

Overall, the currently available data indicate that DVL expression and function have diverged to some degree apparently and consistent with phylogenetic models mostly between DVL1 and DVL2/3. Thus, depending on the cellular context, DVL paralogs exhibit both redundant and distinct functionality.

## Author contributions

All authors listed, have made substantial, direct and intellectual contribution to the work, and approved it for publication.

### Conflict of interest statement

The authors declare that the research was conducted in the absence of any commercial or financial relationships that could be construed as a potential conflict of interest.
