# Supplementary material for: Dishevelled Paralogs in Vertebrate Development: Redundant or Distinct?
Source: Front Cell Dev Biol. 2017 May 26;5:59. doi: 10.3389/fcell.2017.00059 (PMC5445114; doi:10.3389/fcell.2017.00059)
Supplement: Supplementary file 1 [file Table1.pdf]

Supplementary Table 1: Overview of DVL knock-out mouse phenotypes

Genotype / Phenotype	***DVL1***$^{-/-}$	***DVL2***$^{-/-}$	***DVL3***$^{-/-}$	***DVL1***$^{-/-}$***;*** ***DVL2***$^{-/-}$	***DVL2***$^{+/-}$***;*** ***DVL3***$^{-/-}$	***DVL2***$^{-/-}$***;*** ***DVL3***$^{+/-}$	***DVL2***$^{-/-}$***;*** ***DVL3***$^{-/-}$	***DVL1***$^{-/-}$***;*** ***DVL3***$^{-/-}$	***DVL1***$^{-/-}$***;*** ***DVL2***$^{+/-}$***;*** ***DVL3***$^{-/-}$	***DVL1***$^{-/-}$***;*** ***DVL2***$^{-/-}$***;*** ***DVL3***$^{+/-}$	***DVL1***$^{-/-}$***;*** ***DVL2***$^{-/-}$***;*** ***DVL3***$^{-/-}$
abnormal social behavior	+										
sensorimotor gating defects	+										
neuronal differentiation and function	+										
congenital heart defects		+	+	+	+	+					
skeletal defects posterior					+	+					
skeletal defects vertebrae and ribs		+		+							
neural tube defects		+ (2-3%)		+ (>90%)	+	+					
PCP defect in organ of Corti			+	+	+						
Nodal cilia defects									+	+	
cilia defects in ependymal cells (hydrocephalus)										+ (cond.)	
gastrulation defects											+
anterior-posterior shortening				+					+		
embryonic lethal						+ (E9.5)	+ (E8.5)	+ (E13.5-15.5)	+	?	+

Ahmad-Annuar, A., Ciani, L., Simeonidis, I., Herreros, J., Fredj, N. B., Rosso, S. B., et al. (2006). Signaling across the synapse: a role for Wnt and Dishevelled in presynaptic assembly and neurotransmitter release. *J Cell Biol* 174, 127–139. doi:10.1083/jcb.200511054.

Hamblet, N. S., Lijam, N., Ruiz-Lozano, P., Wang, J., Yang, Y., Luo, Z., et al. (2002). Dishevelled 2 is essential for cardiac outflow tract development, somite segmentation and neural tube closure. *Development* 129, 5827–5838. doi:10.1242/dev.00164

Hashimoto, M., Shinohara, K., Wang, J., Ikeuchi, S., Yoshiba, S., Meno, C., et al. (2010). Planar polarization of node cells determines the rotational axis of node cilia. *Nat Cell Biol* 12, 170–176. doi:10.1038/ncb2020.

Lijam, N., Paylor, R., McDonald, M. P., Crawley, J. N., Deng, C. X., Herrup, K., et al. (1997). Social interaction and sensorimotor gating abnormalities in mice lacking Dvl1. *Cell* 90, 895–905. doi:10.1016/S0092-8674(00)80354-2

Ohata, S., Nakatani, J., Herranz-Pérez, V., Cheng, J., Belinson, H., Inubushi, T., et al. (2014). Loss of Dishevelleds Disrupts Planar Polarity in Ependymal Motile Cilia and Results in Hydrocephalus. *Neuron* 83, 558–571. doi:10.1016/j.neuron.2014.06.022.

Rosso, S. B., Sussman, D., Wynshaw-Boris, A., and Salinas, P. C. (2005). Wnt signaling through Dishevelled, Rac and JNK regulates dendritic development. *Nat Neurosci* 8, 34–42. doi:10.1038/nn1374.

Sinha, T., Wang, B., Evans, S., Wynshaw-Boris, A., and Wang, J. (2012). Disheveled mediated planar cell polarity signaling is required in the second heart field lineage for outflow tract morphogenesis. *Dev Biol* 370, 135–144. doi:10.1016/j.ydbio.2012.07.023.

Wang, J., Hamblet, N. S., Mark, S., Dickinson, M. E., Brinkman, B. C., Segil, N., et al. (2006). Dishevelled genes mediate a conserved mammalian PCP pathway to regulate convergent extension during neurulation. *Development* 133, 1767–1778. doi:10.1242/dev.02347.

Wang, J., Mark, S., Zhang, X., Qian, D., Yoo, S.-J., Radde-Gallwitz, K., et al. (2005). Regulation of polarized extension and planar cell polarity in the cochlea by the vertebrate PCP pathway. *Nat Genet* 37, 980–985. doi:10.1038/ng1622.
